# Synthetic lethality of cytolytic HSV-1 in cancer cells with ATRX and PML deficiency

**DOI:** 10.1242/jcs.222349

**Published:** 2019-03-14

**Authors:** Mingqi Han, Christine E. Napier, Sonja Frölich, Erdahl Teber, Ted Wong, Jane R. Noble, Eugene H. Y. Choi, Roger D. Everett, Anthony J. Cesare, Roger R. Reddel

**Affiliations:** 1 Cancer Research Unit, Children's Medical Research Institute, Faculty of Medicine and Health, The University of Sydney, Westmead, NSW 2145, Australia; 2 Genome Integrity Unit, Children's Medical Research Institute, Faculty of Medicine and Health, The University of Sydney, Westmead, NSW 2145, Australia; 3 Bioinformatics Group, Children's Medical Research Institute, Faculty of Medicine and Health, The University of Sydney, Westmead, NSW 2145, Australia; 4 MRC–University of Glasgow Centre for Virus Research, Bearsden, Glasgow G61 1QH, Scotland, UK

**Keywords:** Sarcoma, Soft-tissue malignancies, Telomeres, Telomerase, Post-transcriptional control, Translational control, Oncolytic virus, ATRX, PML

## Abstract

Cancers that utilize the alternative lengthening of telomeres (ALT) mechanism for telomere maintenance are often difficult to treat and have a poor prognosis. They are also commonly deficient for expression of ATRX protein, a repressor of ALT activity, and a component of promyelocytic leukemia nuclear bodies (PML NBs) that are required for intrinsic immunity to various viruses. Here, we asked whether ATRX deficiency creates a vulnerability in ALT cancer cells that could be exploited for therapeutic purposes. We showed in a range of cell types that a mutant herpes simplex virus type 1 (HSV-1) lacking ICP0, a protein that degrades PML NB components including ATRX, was ten- to one thousand-fold more effective in infecting ATRX-deficient cells than wild-type ATRX-expressing cells. Infection of co-cultured primary and ATRX-deficient cancer cells revealed that mutant HSV-1 selectively killed ATRX-deficient cells. Sensitivity to mutant HSV-1 infection also correlated inversely with PML protein levels, and we showed that ATRX upregulates PML expression at both the transcriptional and post-transcriptional levels. These data provide a basis for predicting, based on ATRX or PML levels, which tumors will respond to a selective oncolytic herpesvirus.

## Abstract (precis)

**Summary:** ATRX deficiency in cancer cells induces downregulation of PML, rendering the cells highly sensitive to lysis with ICP0-null mutant herpes simplex virus-1, with potential therapeutic applications.

## INTRODUCTION

Healthy cells can divide a limited number of times, whereas cancer cell populations usually acquire an unlimited proliferative capacity. Telomeres are the nucleoprotein structures at the termini of chromosomes, which, due to the end-replication problem, shorten with each cell division. Most human tumors activate a telomere lengthening mechanism, either telomerase (TEL) or alternative lengthening of telomeres (ALT), to counteract telomere shortening and thereby enable unlimited cellular proliferation (Kim et al., 1994; Bryan et al., 1995). Approximately 85–90% of tumors activate telomerase, usually through dysregulated expression of its catalytic component, telomerase reverse transcriptase (TERT), which is caused, for example, by activating mutations in the promoter region of the *TERT* gene (Shay and Bacchetti, 1997; Zhang et al., 2000a; Horn et al., 2013; Huang et al., 2013). ALT is activated in many of the remaining 10–15% of cancers, and is common in various cancers including osteosarcomas, several soft tissue sarcoma subtypes, and astrocytomas including pediatric glioblastoma (Bryan et al., 1997; Henson et al., 2005; Heaphy et al., 2011b). Loss of the chromatin remodeling protein α-thalassemia/mental retardation syndrome X-linked (ATRX) or its heterodimeric binding partner, death domain-associated protein 6 (DAXX) have been identified in a significant proportion of tumors and cell lines that utilize ALT (Heaphy et al., 2011a; Bower et al., 2012; Jiao et al., 2012; Lovejoy et al., 2012).

ATRX and DAXX are constitutive components of promyelocytic leukemia nuclear bodies (PML NBs), and these subnuclear structures are indispensable for intrinsic immunity (Xue et al., 2003; Bieniasz, 2004). PML NBs act as a first line of defense against viral infection, specifically by associating with and silencing viral genes (Tavalai and Stamminger, 2008). Incomplete PML NBs generated by knockdown of one or more constitutive PML NB proteins, such as PML, SP100, ATRX or DAXX, resulted in loss of the ability of human cells to hinder wild-type herpes simplex type 1 (WT HSV-1) replication (Everett et al., 2006, 2008; Lukashchuk and Everett, 2010; Glass and Everett, 2013). The HSV-1 immediate early protein ICP0, which is an E3 ubiquitin ligase (Boutell and Everett, 2003; Lilley et al., 2010), is involved in counteracting the intrinsic immunity qualities of PML NBs, and ICP0-null HSV-1 proliferates very poorly in cells with intact PML NBs (Stow and Stow, 1986; Cai and Schaffer, 1989). However, disruption of PML NBs by knockdown of ATRX alone, DAXX alone, DAXX and PML, or DAXX, PML and SP100, facilitates replication of ICP0-null HSV-1 (Everett et al., 2008; Lukashchuk and Everett, 2010; Glass and Everett, 2013).

Here, we have investigated whether the deficiency of ATRX protein expression that is common in ALT-dependent cancers creates an opportunity for a synthetic-lethal treatment strategy (Kaelin, 2005). Specifically, we asked whether ICP0-null HSV-1, which is unable to effectively infect cells with intact PML NBs, is able to infect and kill ATRX-deficient cancer cells. We found that infectivity of the mutant virus was 10- to 1,000-fold greater in ATRX-deficient cells than in ATRX-positive cells, and also in cells with low expression of PML protein. Moreover, we found for the first time that ATRX regulates PML expression, and that this occurs at both the transcriptional and post-transcriptional levels. These data indicate that ATRX and/or PML levels could be used to predict response to this oncolytic virus.

## RESULTS

### ATRX deficiency enhances infectivity of ICP0-null HSV-1

**Fig. 1.**
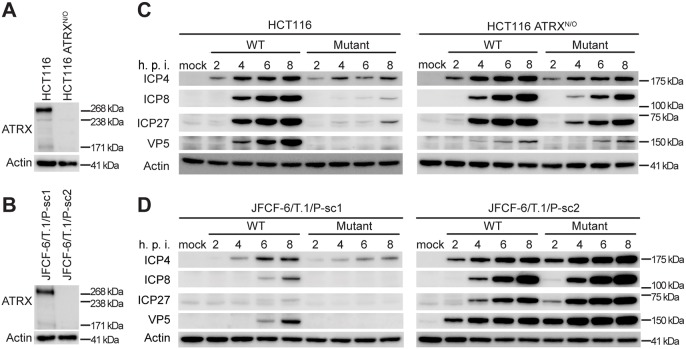
**Loss of ATRX in** **infected cells increases expression of mutant HSV-1 viral genes.** (A,B) ATRX protein expression evaluated using western blotting in two cell line pairs: wild-type HCT116 and ATRX-knockout HCT116 ATRX^N/O^ (A), and JFCF-6/T.1/P-sc1 (ATRX-positive) and JFCF-6/T.1/P-sc2 (ATRX-deficient) (B). (C,D) Expression of viral proteins during infection. The cell line pairs were infected with WT or mutant HSV-1, and harvested at the indicated times (h.p.i., hours post-infection). The antibodies used for viral protein detection are indicated to the left of each panel.

Intrinsic immunity to viral infection involves translocation of PML NB components to the nuclear periphery to inhibit viral replication (Everett and Murray, 2005). Using an HSV-1 mutant strain with an inactivating deletion in ICP0, we compared the infectivity of wild-type (WT) and ICP0-null (mutant) HSV-1 in two pairs of closely-related cell lines. One pair consisted of a TEL-positive cell line (HCT116) and its subline generated by inactivating ATRX by gene targeting (HCT116 ATRX^N/O^) (Fig. 1A). The other pair of cell lines was derived from one fibroblast line by two different spontaneous immortalization events, with one being an ALT-positive cell line containing a spontaneous inactivating mutation in ATRX (JFCF-6/T.1/P-sc1), and the other being a TEL-positive line expressing ATRX (JFCF-6/T.1/P-sc2) (Fig. 1B). We found that expression of viral proteins, including immediate early proteins involved in replication compartment assembly (ICP4, ICP8 and ICP27) and the capsid protein expressed at late stage (VP5), was strongly limited in ATRX-expressing cells infected with mutant HSV-1 as compared to WT HSV-1 (Fig. 1C,D, left panels). In contrast, WT and mutant virus produced similar levels of viral proteins in cells lacking ATRX (Fig. 1C,D, right panels).

**Fig. 2.**
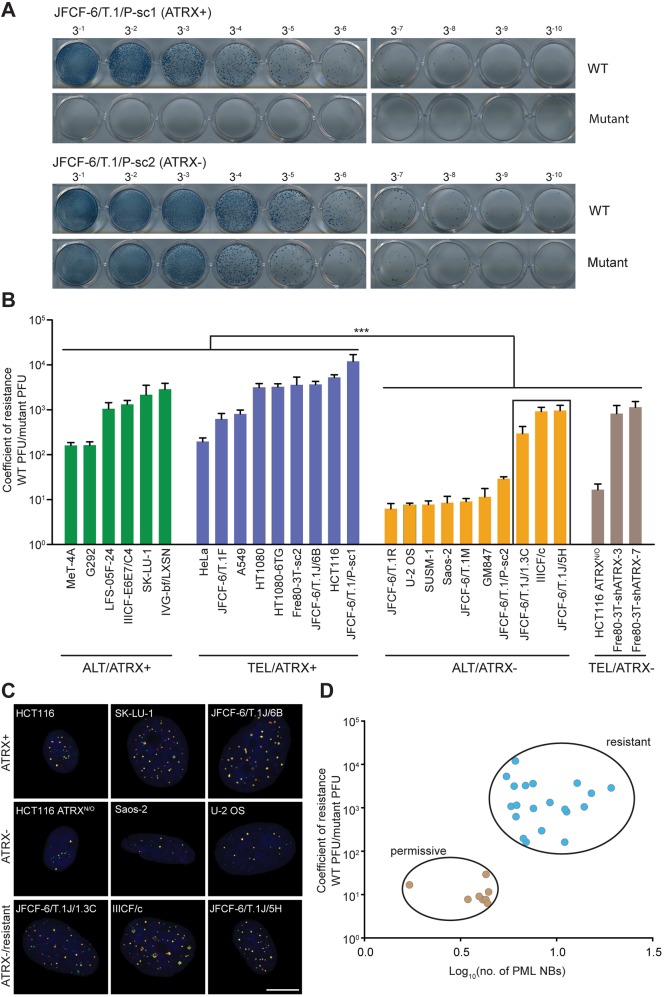
**ATRX expression and PML NB count correlate with sensitivity to mutant HSV-1.** (A) Representative images of plaque-forming assays used to calculate the coefficient of resistance. Indicated cell lines were infected with serially diluted viral preparations (1 in 3 dilutions from left to right). Infected cells express β-galactosidase and plaques were detected by X-gal staining. (B) Cell lines were grouped as either ATRX-positive (ATRX+) or ATRX-deficient (ATRX−), in combination with either telomerase (TEL) or alternative lengthening of telomeres (ALT) telomere maintenance characteristics. Viral plaques were counted 30 h after infection of the indicated cell lines and the coefficient of resistance (WT PFU/mutant PFU) was plotted. PFU, plaque-forming unit. Error bars: mean±s.e.m. of three biologic replicates; ***P<0.001, Mann–Whitney test grouping ATRX-positive versus ATRX-deficient cell lines. The box around three ALT/ATRX− cell lines indicates the resistant cell lines. (C) Representative images of PML NB staining in ATRX-positive, ATRX-deficient and infection-resistant ATRX-deficient (ATRX−/resistant) cells. PML protein, red; SP100 protein, green; DNA counterstained with DAPI, blue. Scale bar: 10 μm. (D) Coefficient of resistance segregates with PML NB number/cell. Data represent mean values for individual cell lines from three biologic replicates. Permissive cell lines are defined as having <6 PML NBs per cell and a coefficient of resistance <60, whereas resistant cell lines have >6 PML NBs per cell and a coefficient of resistance >60.

To determine the impact of ICP0-null HSV1 infection on cell viability, we determined plaque formation in a panel of cell lines, including the two cell line pairs discussed above (Fig. 2A,B). Each cell line's coefficient of resistance was calculated as the ratio of plaques formed following infection with WT versus mutant virus. The cell line panel comprised WT ATRX-expressing and ATRX-deficient cell lines that were further subcategorized by telomere length maintenance mechanism (ALT or TEL; Table S1). ATRX-deficient cells were 10 to 1000 times less resistant to mutant HSV-1 infection than WT ATRX-expressing cells, regardless of telomere length maintenance mechanism. However, of ten ATRX-deficient ALT cell lines, three lines (JFCF-6/T.1J/1.3C, IIICF/c and JFCF-6/T.1J/5H) exhibited resistance to viral infection that was >60-fold greater than the remaining seven. On average, however, these three ATRX-deficient ALT cell lines were four times less resistant than the ATRX-positive panel of 15 cell lines.

We analyzed the cell cycle profile of the ATRX-positive and -negative cancer cell line pair, HCT116 and HCT116 ATRX^N/O^, growing exponentially and asynchronously, and found similar cell cycle profiles (percentages of cells in the G_1_, S and G_2_/M compartments were 37.8±0.4, 28.1±0.3, 31.5±0.5 for HCT116 cells; and 34.4±0.5, 27.3±0.5 and 33.9±1.1 for HCT116 ATRX^N/O^ cells; mean±s.e.m., *n*=3). Thus, there was no evidence that a change in cell cycle parameters accounted for the difference in anti-viral resistance following knock-out of the *ATRX* gene in this cell line.

### PML NB count correlates with resistance to mutant HSV-1

Because ATRX is a constitutive component of PML NBs, we examined these nuclear structures in the cell line panel. Automated quantitation of PML NBs using immunofluorescence staining of PML and SP100 proteins revealed that the resistant ATRX-deficient/ALT cell lines contain more PML NBs than cell lines that were sensitive to mutant virus infection (Fig. 2C; Fig. S1A). A plot of the coefficient of resistance against the number of PML NBs/cell shows a clear distinction between cell lines permissive to infection (defined here as having a coefficient of resistance <60) and a smaller number of PML NBs versus cell lines that are resistant to infection and have a greater PML NB count (Fig. 2D). These data demonstrate that resistance to the mutant virus infection segregates with the number of PML NBs.

### Inactivation of ATRX diminishes cellular PML levels

**Fig. 3.**
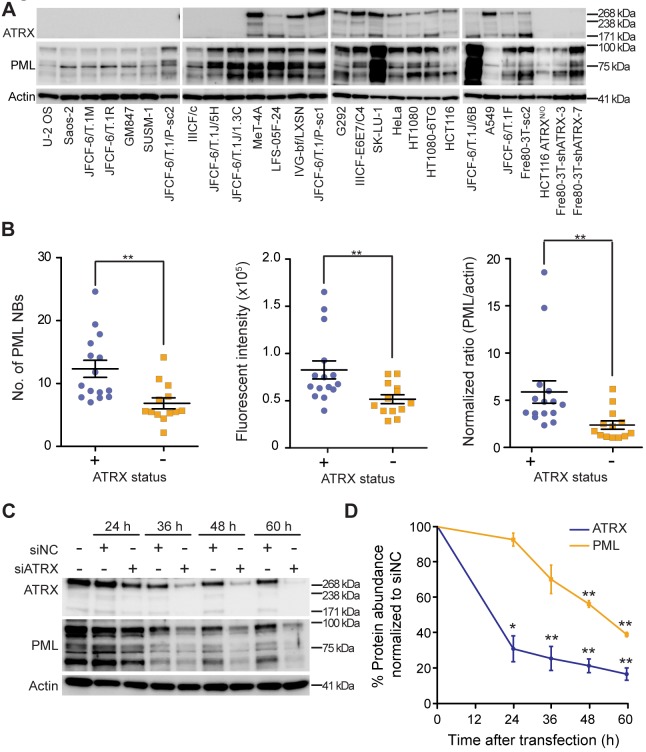
**ATRX status correlates with PML expression levels.** (A) ATRX and PML protein expression was analyzed by means of western blot in the indicated panel of cell lines. (B) PML NBs per cell (left plot) and PML fluorescence intensity within PML NBs (center plot) were quantitated using automated imaging. PML protein expression was also quantitated by western blot and plotted relative to actin and normalized to the JFCF-6/T.1M cell line (right plot). Each data point represents a cell line, >200 nuclei counted per cell line, mean±s.e.m., *n*=3 independent experiments. Cell lines are grouped by ATRX status; **P<0.01, Mann–Whitney test. (C) HT1080 cells were treated with control siRNA (siNC) or siATRX for the time indicated, and western blotting performed at the indicated timepoints to evaluate expression levels of ATRX and PML. (D) Quantitation of ATRX and PML expression subsequent to siNC or siATRX transfection, as shown in C. Expression was normalized to actin, and then to the siNC-treated sample at each time point. Data are expressed as the mean±s.e.m. of three biologic replicates; *P<0.05, **P<0.01, paired two-tailed *t*-test.

We further investigated the relationship between ATRX, PML and PML NBs and found that the number of PML NBs, the intensity of PML immunofluorescence staining inside PML NBs, and PML protein expression as determined by western blot significantly correlated with ATRX status (Fig. 3A,B; Fig. S1B). For the majority of ATRX-positive cell lines, there was a strong correlation between ATRX and PML levels. The correlation for all cell lines was R^2^=0.05 (Fig. S1C), but when two outliers (SK-LU-1 and JFCF-6/T.1J/6B) with high ATRX expression and one (MeT-4A) with high PML were removed, R^2^=0.75 (Fig. S1D). To determine if the relationship between ATRX and PML expression is causal, we depleted ATRX in HT1080 fibrosarcoma cells using siRNA treatment and examined PML protein expression. PML protein levels were significantly decreased after ATRX depletion relative to control cells (Fig. 3C,D; Fig. S1E), demonstrating that ATRX positively regulates PML expression. Furthermore, we confirmed this result in five ATRX-positive cell lines, including three ALT cell lines (SK-LU-1, IIICF-E6E7/C4 and G292) and two TEL cell lines (HeLa and Fre80-3T-sc2) (Fig. S2A). Consistent with the result demonstrated in HT1080 cells, depletion of ATRX reduced PML protein levels by up to 50% when compared to control siRNA­-treated samples (Fig. S2B).

To determine whether ATRX loss of function affects the subset of PML NBs that have telomeric content [i.e. ALT-associated PML bodies (APBs)], we quantitated APBs in three ATRX-positive (SK-LU-1, IIICF-E6E7/C4 and IVG-bf/LXSN) and three ATRX-deficient (GM847, IIICF/c and Saos-2) cell lines (>600 nuclei in each group). The number of APBs per nucleus was 3.97±0.14 for ATRX-positive and 3.79±0.14 for ATRX-deficient cell lines (mean±s.e.m.). Therefore, we found no evidence that ATRX status affects APB numbers.

### ATRX regulates PML expression at the level of both transcription and protein stability

**Fig. 4.**
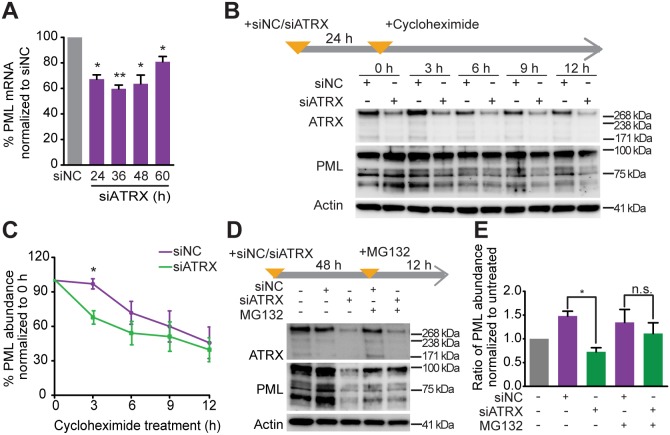
**ATRX regulates PML expression at the transcriptional and post-transcriptional levels.** (A) HT1080 cells were depleted of ATRX through treatment with siATRX for the indicated times. Real-time PCR analysis of PML expression was performed on samples from the indicated time points. Data were normalized to values from siNC-treated cells. Data are expressed as the mean±s.e.m., *n*=3 independent experiments. *P<0.05, **P<0.01, paired two-tailed *t*-test. (B,C) HT1080 cells were treated with siNC or siATRX for 24 h, followed by cycloheximide (30 ng µl^−1^) for the indicated times. Expression of ATRX and PML was analyzed by western blot (B). Data (mean±s.e.m.) are plotted as the ratio of PML:actin expression, and the ratio of each time point was then normalized to the non-cycloheximide control (0 h) (C). *P<0.05, paired two-tailed *t*-test. (D,E) HT1080 cells were treated with siNC, siATRX and/or MG132 (5 µM) as indicated above each lane (D). PML:actin expression ratio was calculated and normalized to the untreated control (E). n.s., not significant, *P<0.05; paired two-tailed *t*-test.

We next investigated the mechanism whereby ATRX regulates PML, and found that PML transcription was reduced in response to treatment with siRNA targeting *ATRX* (siATRX) (Fig. 4A). Assaying PML degradation kinetics using the protein synthesis inhibitor cycloheximide showed that PML protein was degraded more rapidly in siATRX-treated HT1080 cells as compared to control cells (Fig. 4B,C). Furthermore, inhibition of the proteasome with MG132 in siATRX-treated HT1080 cells partially stabilized PML protein (Fig. 4D,E). These data indicate that ATRX regulates PML expression, and protects PML protein from proteasome-dependent degradation, thus resulting in the accumulation of PML protein in control cells. Taken together, these results indicate that ATRX controls PML expression at both the transcriptional and post-translational level.

### Modification of ATRX and/or PML expression influences sensitivity to mutant HSV-1

**Fig. 5.**
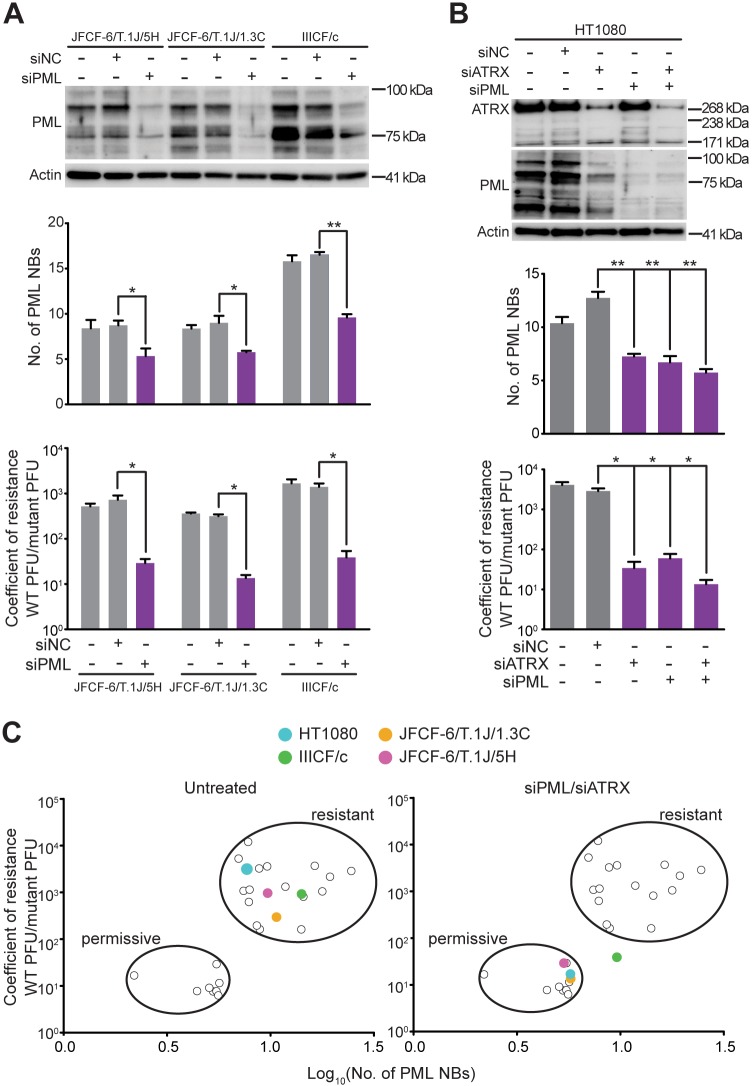
**Depletion of PML increases sensitivity to cytolytic virus.** (A,B) The three virus-resistant ALT cell lines (A) and the HT1080 (TEL) cell line (B) were treated with siNC or siPML (and/or siATRX for HT1080) for 72 h, and depletion of PML was confirmed by western blot. Graphs show PML NB number (top) and coefficient of resistance to viral infection (bottom); mean±s.e.m., *n*=3 independent experiments; *P<0.05, **P<0.01 using a paired two-tailed *t*-test. (C) Coefficient of resistance versus PML NB numbers was plotted for untreated and PML and/or ATRX-depleted (siPML/siATRX) cells, with the three resistant ALT lines and HT1080 cells indicated in colored circles; the remaining data points are as previously shown in Fig. 2D.

We next determined whether manipulating PML expression altered the viral resistance of the three ATRX-deficient ALT cell lines with elevated numbers of PML NBs (JFCF-6/T.1J/5H, JFCF-6/T.1J/1.3C and IIICF/c). As expected, treatment with siRNA targeting *PML* (siPML) reduced PML protein expression, and also decreased the number of PML NBs and significantly reduced the resistance of these cell lines to mutant HSV-1 infection (Fig. 5A; Fig. S3A,B). To further confirm our findings that PML and ATRX expression are intimately linked to mutant HSV-1 resistance, we depleted PML and/or ATRX in HT1080 cells. Depletion of either, or both, proteins caused a significant reduction in PML NB numbers and a significantly reduced resistance to mutant HSV-1 infection (Fig. 5B; Fig. S3C). These results further demonstrate that cellular PML NB levels correlate with resistance to mutant HSV-1 infection (Fig. 5C).

### Healthy cells and ATRX-expressing fibroblasts are resistant to mutant HSV-1

**Fig. 6.**
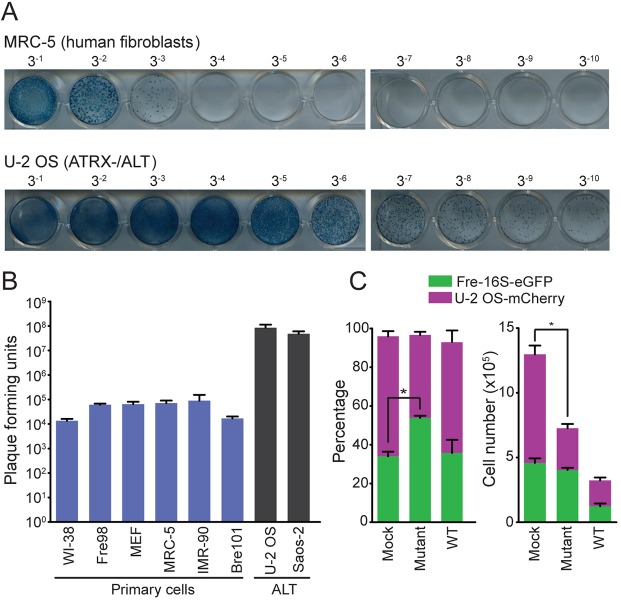
**Mutant HSV-1 is selectively toxic to ATRX-deficient cells.** (A) Plaque assays for MRC–5 human fibroblasts and U-2 OS (ATRX-deficient/ALT) cells infected with a 1:3 serial dilution of mutant HSV-1. (B) Primary (WI-38, Fre98, MEF, MRC-5, IMR-90 and Bre101) and ALT-positive (U-2 OS and Saos-2) cells were infected with mutant HSV-1; numbers of plaque forming units are shown as mean±s.e.m., *n*=3 independent experiments. (C) eGFP-labeled fibroblasts (Fre-16 s) and U-2 OS–mCherry cells were mixed in a ratio that yielded an equivalent number of cells 12 h after plating, and then infected with WT or mutant HSV-1 at an MOI of 0.96. The percentage (left plot) and the number (right plot) of eGFP- and mCherry-labeled cells remaining 30 h after mock or viral infection as determined by FACS is graphed as mean±s.e.m., *n*=3 independent experiments. *P<0.05, paired two-tailed *t*-test.

To gain insight into the feasibility of using the mutant HSV-1 as a therapy for ATRX-deficient tumors, we confirmed that mutant HSV-1 replicates ∼1000-fold more effectively in ATRX-deficient tumor cells than in ATRX-expressing fibroblast or healthy epithelial cells (Fig. 6A,B). We obtained further evidence that mutant HSV-1 selectively targets ATRX-deficient cells through infecting fluorescently tagged ATRX-expressing and ATRX-deficient cell co-cultures with WT or mutant HSV-1. Mutant HSV-1 infection resulted in a 61% decrease (*P*=0.016) in the number of ATRX-deficient cells after only 36 h, whereas the effect on ATRX-expressing cells was not significant (Fig. 6C; Fig. S4). In contrast, WT HSV-1 infection caused equivalent decreases in the numbers of ATRX-deficient and ATRX-expressing cells. These data provide conclusive evidence that mutant HSV-1 replicates more effectively in ATRX-deficient cells than in ATRX-expressing cells.

## DISCUSSION

Here, we have uncovered additional complexity in the relationship between the PML and ATRX proteins and the ALT mechanism. ALT is an homologous recombination-dependent, break-induced telomere synthesis mechanism (Reddel et al., 1997; Dunham et al., 2000; Dilley et al., 2016; Garcia-Exposito et al., 2016), and there is some evidence that PML NBs may play a role in this process (Pickett and Reddel, 2015). A subset of the PML NBs in ALT cells contain telomeric DNA, some of which is extrachromosomal, together with shelterin proteins and proteins involved in homologous recombination. Because these PML NBs are highly characteristic of cancer cells that use ALT, they are referred to as ALT-associated PML bodies (APBs) (Yeager et al., 1999). It has been observed that telomeres move into PML bodies in ALT cells (Molenaar et al., 2003) and that this is enhanced by telomere DNA breaks (Cho et al., 2014). PML is thought to promote clustering and recombination of telomeres within APBs (Draskovic et al., 2009; Chung et al., 2011). Moreover, a common genetic change in ALT cancers and cell lines is a loss-of-function mutation in ATRX (Heaphy et al., 2011a; Bower et al., 2012; Jiao et al., 2012; Lovejoy et al., 2012), which is normally a constitutive component of PML NBs (Xue et al., 2003; Bieniasz, 2004). This is consistent with ATRX being a suppressor of ALT (Clynes et al., 2015; Napier et al., 2015).

The mechanism whereby ATRX suppresses ALT is unknown, but one speculation is that loss of ATRX function allows PML NBs to participate in telomere lengthening, whereas this function is suppressed when ATRX is present. We were therefore initially surprised to find here that the number and size of PML foci, and the PML protein content, were reduced in ATRX-deficient ALT cells. We found that the decrease in PML levels was a direct result of the loss of ATRX-mediated upregulation of PML levels at both the transcriptional and post-translational levels. Moreover, we found no correlation between ATRX status and number of APBs per nucleus. It is therefore clear that the reduced level of PML resulting from loss of ATRX function is compatible with APB formation and ALT activity. Three of the ATRX-deficient cell lines (JFCF-6/T.1J/1.3C, IIICF/c and JFCF-6/T.1J/5H) did not have a reduction in PML levels. Moreover, the correlation coefficient for PML and ATRX in ATRX-positive cell lines is consistent with the hypothesis that, in addition to ATRX, other factors contribute to the control of PML levels.

Although investigating mechanisms apart from ATRX for controlling PML levels is outside the scope of this study, we looked for loss-of-function mutations in E3 ubiquitin ligase genes in these ATRX-deficient cell lines that could potentially result in a reduced rate of PML degradation. Sequencing of the *CBX4*, *UBE3A*, *HECTD1*, *HECTD2*, *MDM2*, *PARK2*, *PIAS1*, *PIN1*, *RNF4* and *SMURF1* genes in these three cell lines identified a deletion of 160,305 bp in *PARK2* in JFCF-6/T.1J/1.3C and RNA-seq showed that this was associated with a threefold decrease in *PARK2* expression (data not shown). *PARK2* expression was also reduced (twofold) in IIICF/c cells. Other factors that could counterbalance the effect of ATRX loss on PML levels potentially include upregulated JAK-STAT signaling (Hubackova et al., 2012).

Many tumors that depend on ALT are difficult to treat and have a poor prognosis (Henson and Reddel, 2010). Given the association between ALT and ATRX loss-of-function mutations, and the role of ATRX in intrinsic resistance to viral infection, we examined whether this difference between healthy cells and ALT tumor cells creates a vulnerability in ALT cancers that could be exploited for synthetic lethality. We found, as expected, that ATRX deficiency results in selective sensitivity to infection with ICP0-null HSV-1. Another mutant HSV-1, talimogene laherparepvec, was approved by the FDA in 2015 for the treatment of advanced melanoma (Andtbacka et al., 2015), and is in clinical trials for other solid tumors including of the bladder, brain, breast, bronchus, colon, head and neck, liver, ovary, pancreas, rectum and skin (melanoma and non-melanoma, including Merkel cell carcinoma) and soft tissue sarcomas (https://clinicaltrials.gov/, accessed 15 December 2018). The testing done to obtain regulatory approval for this modified virus, and the experience gained with its subsequent clinical use, will most likely facilitate the development of other HSV-1 oncolytic viruses as cancer therapeutics.

In addition to inactivating the ICP0 protein, further modifications could be made to HSV-1 to enhance its selectivity. For example, HSV-1 also expresses high levels of microRNA (miR)-H1 during infection, and one of its targets is the 3′ UTR of ATRX. Furthermore, the HSV-1 tegument protein, virion-associated host shutoff (Vhs), which is an endoribonuclease, was shown to efficiently facilitate degradation of ATRX mRNA (Jurak et al., 2012). Mutations in HSV-1 that inactivate gene products such as these could further hamper its ability to replicate in healthy cells, and therefore enhance its selectivity for cytolysis of cells with genetic lesions, resulting in decreased intrinsic immunity.

We also demonstrate here that total PML content and PML NB number correlate with resistance to the mutant virus, and that depleting cells of PML protein decreases this resistance. The relationship between ATRX deficiency and decreased PML in ALT cells was found to be causal: we showed that ATRX upregulates PML by increasing transcription of its mRNA and decreasing its proteasome-mediated degradation. It has been observed in a number of studies that PML expression is completely or substantially lost, via unknown mechanisms, in many tumor types that are not known to be ATRX-deficient (Zhang et al., 2000b; Gurrieri et al., 2004; Lee et al., 2007; Reineke et al., 2008), and a more extensive survey of PML expression in cancer could potentially reveal many more. In contrast, most healthy tissues display high levels of PML protein and PML NB immunostaining (Gurrieri et al., 2004), indicating that healthy tissues should be relatively resistant to ICP0-null HSV-1. Therefore, although the vulnerability to ICP0-null HSV-1 was found through testing a hypothesis regarding ATRX-deficient ALT cancer cells, the additional findings regarding PML may have uncovered a target for selective oncolytic viral therapy in a wider array of tumor types, namely, low PML levels, regardless of the cause of the downregulation.

## MATERIALS AND METHODS

### Cell culture

Baby hamster kidney-21 (BHK21) cells were grown in Glasgow modified Eagle's medium (GMEM; Thermo Fisher Scientific, Melbourne, Australia), 10% fetal bovine serum (FBS; Sigma-Aldrich, Castle Hill, Australia) and 10% tryptose phosphate broth (TPB). TPB consists of 20 g l^−1^ tryptose, 2 g l^−1^ dextrose, 5 g l^−1^ NaCl, 2.5 g l^−1^ disodium phosphate. JFCF-6/T.1R, U-2 OS, SUSM-1, Saos-2, JFCF-6/T.1M, GM847, JFCF-6/T.1/P-sc2, JFCF-6/T.1J/1.3C, IIICF/c, JFCF-6/T.1J/5H, G292, LFS-05F-24, IIICF-E6E7/C4, SK-LU-1, IVG-bf/LXSN, HeLa, JFCF-6/T.1F, A549, HT1080, HT1080-6TG, Fre80-3T-sc2, JFCF-6/T.1J/6B, JFCF-6/T.1/P-sc1, Fre16s, Fre98, MEF, MRC-5 and IMR-90 were cultured in Dulbecco's modified Eagle's medium (DMEM; Thermo Fisher Scientific) supplemented with 10% FBS. MeT-4A cells were grown in DMEM and 5% FBS. Fre80-3T-shATRX-3 and Fre80-3T-shATRX-7 cells were cultured in DMEM, 10% FBS and 0.5 µg ml^−1^ puromycin. HCT116 cells were grown in McCoy's 5A medium (Thermo Fisher Scientific), 10% FBS and 5 mM L-glutamine. HCT116 ATRX^N/O^ cells were grown in McCoy's 5A medium, 10% FBS, and 5 mM L-glutamine supplemented with 1 mg ml^−1^ G418. WI-38 cells were grown in minimum essential medium (Thermo Fisher Scientific), 10% FBS and 5 mM L-glutamine. Bre101 cells were cultured in MCDB 170 medium (Thermo Fisher Scientific). All cell lines were cultured at 37°C in 10% CO_2_ and atmospheric O_2_, with the exception of Bre101 cells, which were grown in 5% CO_2_.

BHK21 cells were obtained from CellBank Australia (Sydney, Australia). The JFCF-6/T- and Fre80-3T-derived cell lines are individual immortalization events following SV40 transfection of a mass population (Lovejoy et al., 2012; Napier et al., 2015). ATRX was inactivated in the HCT116 ATRX^N/O^ cell line using rAAV targeting exon 5 (Napier et al., 2015). All other cell lines were constructed or obtained as described (Huschtscha et al., 2012; Lovejoy et al., 2012; Napier et al., 2015). The identity of all cell lines was confirmed by short tandem repeat DNA analyses at CellBank Australia.

### siRNAs, vectors and antibodies

All siRNAs used in this study were purchased from QIAGEN (Melbourne, Australia). RNAi transfections (40 nM) were performed using Lipofectamine RNAiMax (Thermo Fisher Scientific) using a forward transfection, according to the manufacturer's instructions. The individual siRNA duplexes were: negative control (5′-AATTCTCCGAACGTGTCACGT-3′), PML (5′-AACGACAGCCCAGAAGAGGAA-3′) (Xu et al., 2003), ATRX-5 (5′-ACCGCTGAGCCCATGAGTGAA-3′), ATRX-6 (5′-AGCAGCTACAGTGACGACTAA-3′), ATRX-7 (5′-CCCAGCAATCACAGAAGCCGA-3′) and ATRX-8 (5′-CTCCAGTGCATTTCTATCGTA-3′).

U-2 OS cells were transfected with psi-mH1-mCherry (GeneCopoeia, Rockville, MD, USA) and subjected to long-term culturing in the presence of 0.8 µg ml^−1^ puromycin. U-2 OS cells with mCherry fluorescence within the highest 20% were sorted on a BD Influx at the Westmead Institute for Medical Research (Sydney, Australia) and used for subsequent experiments.

We used primary antibodies raised against: ICP4 (1:300 dilution; Santa Cruz Biotechnology, Tingalpa, Australia, sc-69809), ICP8 (1:500; Abcam, Melbourne, Australia, ab20194), ICP27 (1:300; Santa Cruz, sc69807), VP5 (1:200; Santa Cruz, sc13525), ATRX (1:333; Sigma-Aldrich, HPA001906), PML [Santa Cruz Biotechnology, sc5621 (1:200; western blotting) and sc9862 (1:300; immunofluorescence)], SP100 (1:500; Sigma-Aldrich, HPA016707) and actin (1:1000; Sigma-Aldrich, A2066). The secondary antibodies used were: goat anti-mouse IgG conjugated to horseradish peroxidase (HRP; Dako, Kingsgrove, Australia, P0447), goat anti-rabbit IgG HRP (Dako, P0448), donkey anti-rabbit Alexa Fluor 488 (Thermo Fisher Scientific, A21206) and donkey anti-goat Alexa Fluor 594 (Thermo Fisher Scientific, A11058), all at 1:1000 dilution for both western blotting and immunofluorescence.

### Viral infections and plaque assay

WT HSV-1 (*in*1863) and ICP0-null mutant HSV-1 (*dl*1403) contain the *lacZ* gene under control of the HCMV promoter (Stow and Stow, 1986). Viruses were propagated in BHK21 cells and titrated on U–2 OS cells (Yao and Schaffer, 1995; Everett et al., 2004). For testing viral gene expression, sub-confluent cells were infected with WT or mutant HSV-1 at a multiplicity of infection (MOI) of 2. Cells were agitated every 7 min for 1 h for virus adsorption and then overlaid with GMEM with 10% FBS and 10% TPB. Cells were harvested at the indicated time (h) post­-infection (h.p.i.).

For the plaque assay, cells were seeded in 24-well plates at a density that yielded confluent wells 12 h post-seeding. Cells were infected with sequential threefold dilutions of WT or mutant HSV-1, and the plates were agitated every 7 min for 1 h for virus adsorption and overlaid with medium containing 1% human serum (Lonza, Mt Waverley, Australia). We detected β-galactosidase-positive plaques 30 h after infection using the Senescence-Associated β-Galactosidase Staining Kit (Cell Signaling Technology, Arundel, Australia) following the manufacturer's protocol. The coefficient of resistance was calculated as the ratio of the WT plaque-forming units (PFU) to the mutant PFU.

### Automated PML NB and APB detection and quantification

To visualize PML NBs, cells were seeded on coverslips stained with Alcian Blue (1 mg ml^−1^, Sigma-Aldrich), fixed in 2% paraformaldehyde and further permeabilized with KCM buffer (120 mM KCl, 20 mM NaCl, 10 mM Tris pH 7.5, 0.1% Triton X-100). After blocking for 1 h in antibody dilution buffer (ABDIL: 20 mM Tris pH 7.5, 2% BSA, 0.2% fish gelatin, 150 mM NaCl, 0.1% Triton X-100, 0.1% sodium azide), cells were incubated with anti-PML and anti-SP100 antibodies diluted in ABDIL overnight at 4°C. Following extensive washing with phosphate-buffered saline with 0.1% Tween-20 (PBST), cells were incubated with fluorescently labeled secondary antibodies for 1 h at room temperature, and washed again in PBST. After incubating cells with 50 ng ml^−1^ 4′,6-diamidino-2′-phenylindole hydrochloride (DAPI; D9542, Sigma-Aldrich) for 10 min, cells were mounted on slides with ProLong Gold Antifade Solution (P36930, Thermo Fisher Scientific).

Immunofluorescence with anti-PML and anti-SP100 antibodies had sufficiently low background that quantitation of co-localizing foci was able to be automated using Metafer4 software (Metasystems GmbH, North Ryde, Australia) on an Axioplan 2 microscope (Zeiss, North Ryde, Australia), with a 63× NA (1.4 Plan-Apochromat) oil objective, and appropriate filter cubes (Fig. S5). We captured interphase cells in 10 *z*-planes in 0.25 µm increments. DAPI-stained nuclei were identified and background subtraction, image sharpening and TopHat transformation were applied to the PML and SP100 immunofluorescence channels. PML and SP100 immunofluorescence foci were each identified as foci of >0.2 µm diameter, >20% intensity over background, separated by a minimum distance of 0.5 µm. Co-localizations were events where the center of a PML and an SP100 focus were ≤0.3 µm apart in three dimensions.

APBs were visualized as described for PML NBs, with the modifications being that anti-SP100 antibody staining was omitted, and hybridization to a peptide nucleic acid (PNA) probe was added. PML staining as described above was followed by dehydration of the slides in an ice-cold ethanol series (75% v/v, 85% and 100% ethanol, 2 min each), air drying of the slides, and hybridization with 0.06 ml PNA probe [0.3 µg ml^−1^ Alexa Fluor 488–OO-(CCCTAA)_3_] (F1004, Panagene, Daejeon, Republic of Korea). Slides were heated to 80°C for 12 min, incubated in a humidified chamber overnight at room temperature, rinsed in distilled water, then washed in 1× SSC/50% formamide (15 min, 37°C) and 1× SSC (15 min, 37°C). Slides were rinsed briefly in distilled water, incubated for 5 min at room temperature with DAPI (50 ng ml^−1^), and mounted with ProLong Gold Antifade Solution. A Zeiss Axio Imager was used to acquire images, which were analyzed using CellProfiler image analysis software (Broad Institute, Cambridge, MA, USA). The criterion for automated scoring of an APB was a minimum of 50% overlap between foci detected by PML immunostaining and PNA hybridization.

### Quantitative RT-PCR

RNA was extracted using the QIAGEN RNeasy Mini Kit, and cDNA was synthesized with SuperScript III Reverse Transcriptase (Thermo Fisher Scientific) following standard protocols. cDNA was amplified using FastStart Essential DNA Green Master (Roche, North Ryde, Australia) and analyzed on a Roche LightCycle 96 machine. Gene expression was normalized to GAPDH. Primer sequences were: PML forward 5′-GATGGCTTCGACGAGTTCAA-3′, PML reverse 5′-GGGCAGGTCAACGTCAATAG-3′, GAPDH forward 5′-ACCCACTCCTCCACCTTTG-3′, and GAPDH reverse 5′-CTCTTGTGCTCTTGCTGGG-3′.

### Protein extraction and western blotting

In order to detect viral proteins, total protein was extracted in lysis buffer as described (Bower et al., 2012), and to detect PML and ATRX expression, lysates were prepared by lysing cells in 4× LDS (106 mM Tris-HCl, 141 mM Tris-Base, 2% SDS, 10% glycerol, 0.75% SERVA Blue G50, 0.25% Phenol Red) containing benzonase (Merck Millipore, Bayswater, Australia) and β-mercaptoethanol. Proteins were separated, probed and analyzed as indicated (Bower et al., 2012). PML expression was quantified as the signal between 60 and 100 kDa, covering the predicted molecular mass of PML isoforms localized to the nucleus (Nisole et al., 2013).

### Flow cytometry

Fre-16s-eGFP and U-2 OS-mCherry cells were mixed at a ratio that yielded an equivalent number of cells 12 h after plating, and then infected with WT or mutant HSV–1 at an MOI of 0.96 for 36 h. Cells were fixed in 2% formaldehyde for 10 min at 37°C, chilled on ice for 1 min and washed extensively with FACS buffer (1% FBS in PBS). Samples were analyzed on a BD LSRFortessa (BD, North Ryde, Australia) at the Westmead Institute for Medical Research (Sydney, Australia).

## Supplementary Material

Supplementary information
