# Supplementary material for: Synthetic lethality of cytolytic HSV-1 in cancer cells with ATRX and PML deficiency
Source: J Cell Sci. 2019 Mar 14;132(5):jcs222349. doi: 10.1242/jcs.222349 (PMC6432714; doi:10.1242/jcs.222349)
Supplement: Supplementary information [file joces-132-222349-s1.pdf]

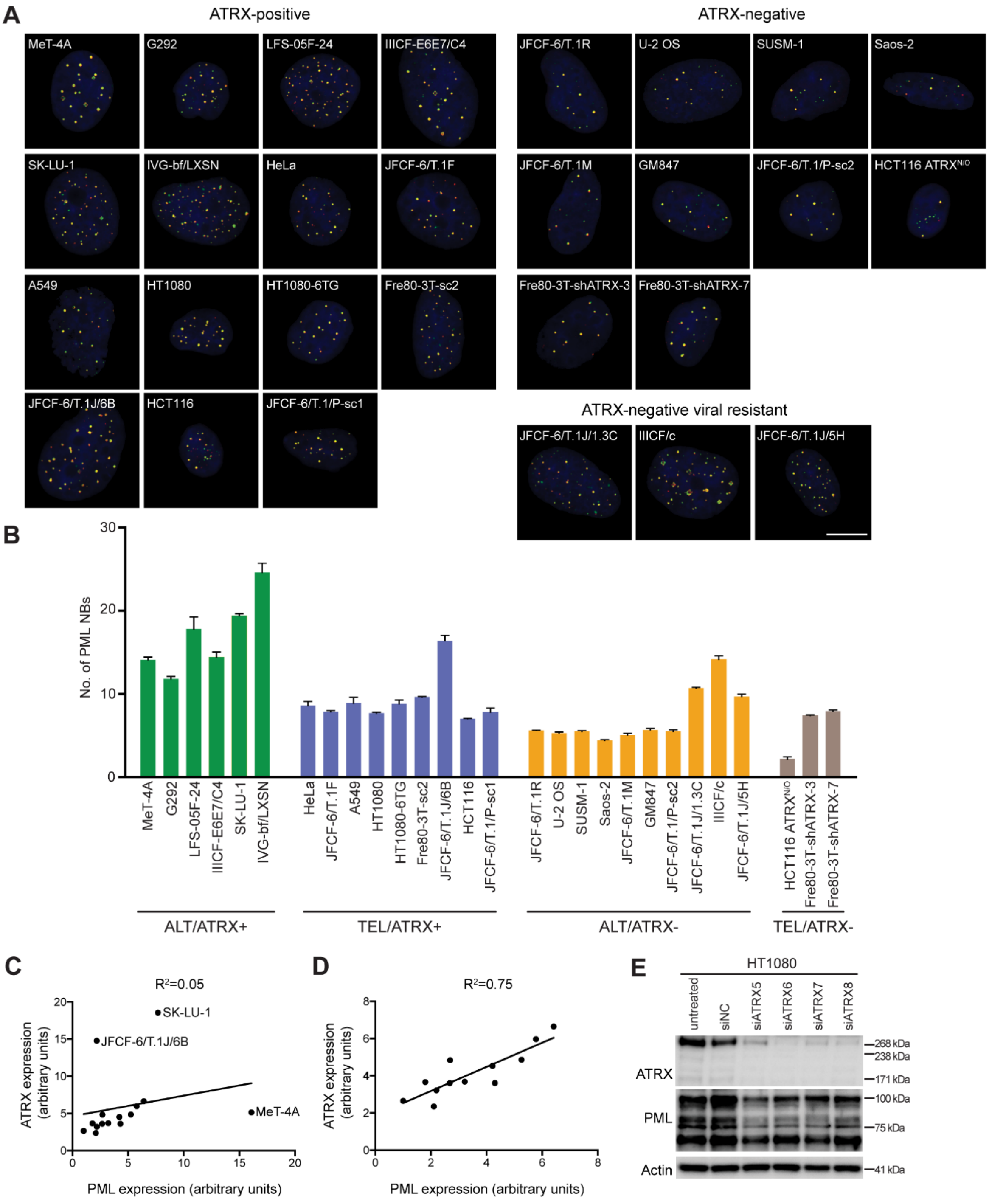

Fig. S1. Correlation between ATRX and PML protein levels. (A) Representative images of PML NB staining for the indicated panel of cell lines. PML protein is stained in red, Sp100 protein in green and DNA counterstained with DAPI in blue. The scale bar denotes 10 µM. (B) The number of PML NBs in the cell line panel was graphed as mean±s.e.m. Data were collected from three independent experiments and >200 nuclei were counted per experiment. PML NB counts were obtained using automated imaging. (C and D) Correlation between ATRX and PML expression in ATRX+ cell lines as quantitated by Western blotting in Fig. 3A and fitted with a linear regression line, with (C) and without (D) data for cell lines with very high ATRX or PML expression. (E) Depletion of ATRX results in decreased PML protein. HT1080 cells were treated with control siRNA (siNC) or four different siATRX sequences for 54 h, and the abundance of ATRX and PML protein was analyzed by Western blot.

Journal of Cell Science • Supplementary information

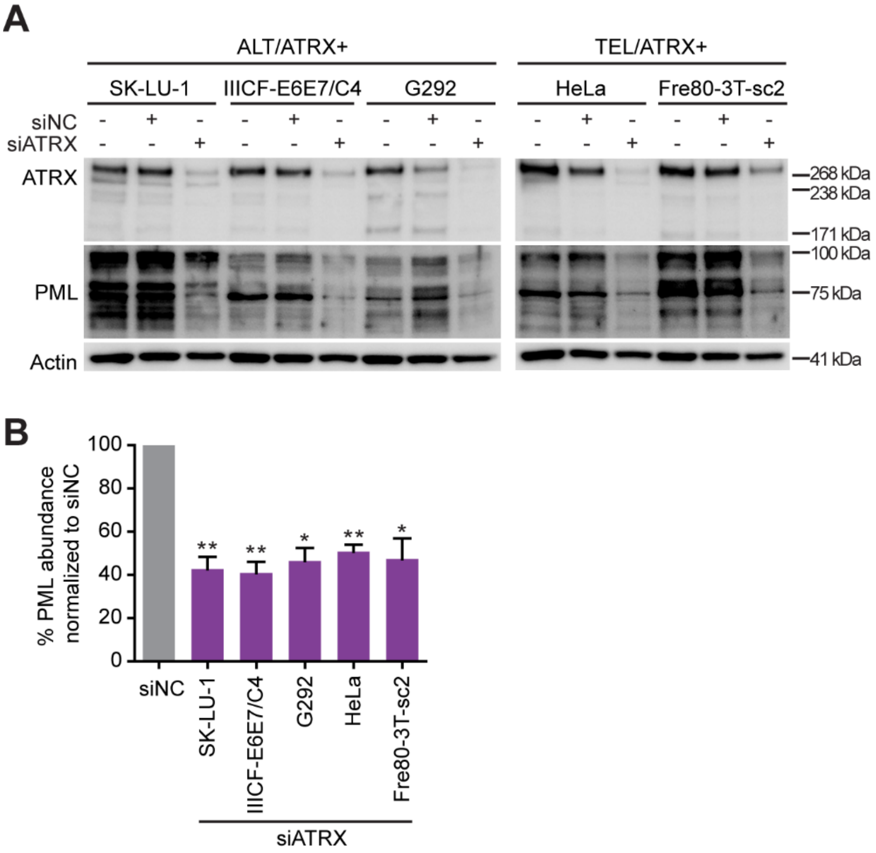

Fig. S2. Alteration in PML levels responding to ATRX knock down. (A) Western blot showing PML, ATRX and actin protein expression in three ALT/ATRX+ cell lines (SK-LU-1, IIICF-E6E7/C4 and G292) and two TEL/ATRX+ cell lines (HeLa and Fre80-3T-sc2) subsequent to treatment with siNC or siATRX for 60 h. (B) Quantification of PML expression, normalized to actin and then the siNC. Bars represent mean±s.e.m. of three independent experiments. *P<0.05, **P<0.01, paired two-tailed t-test.

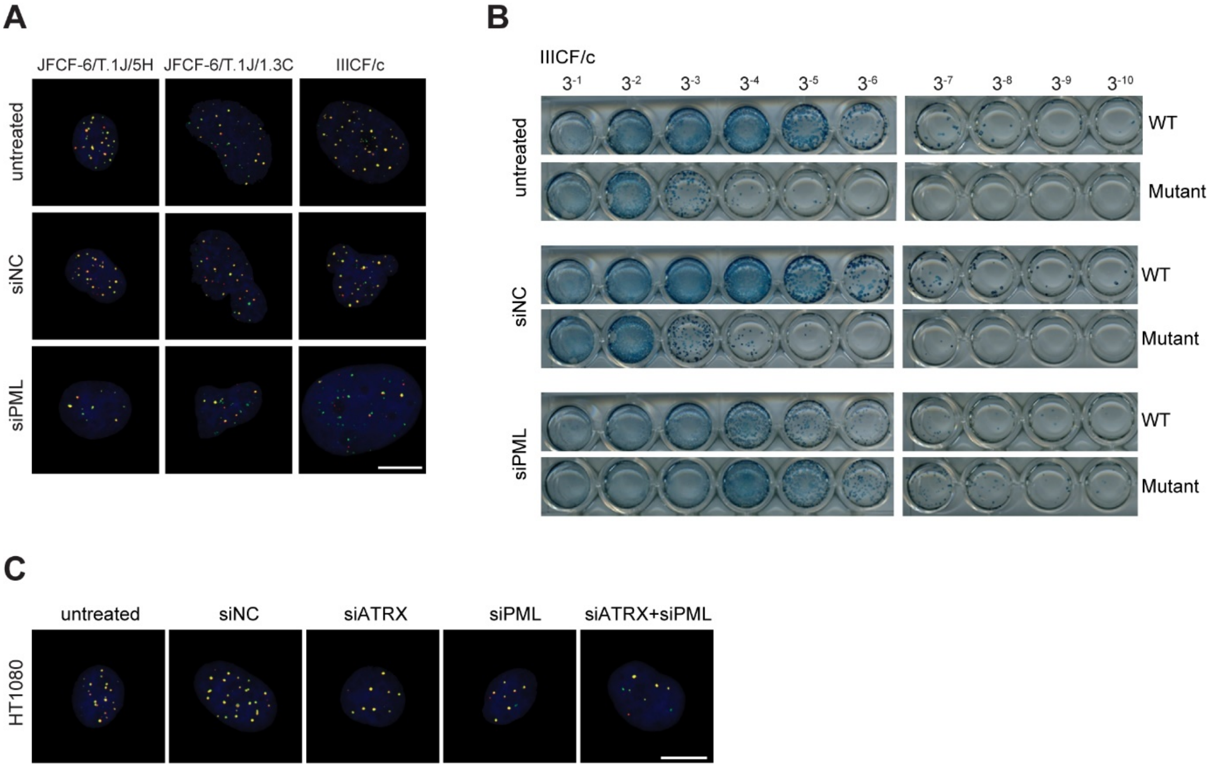

Fig. S3. Depletion of PML increases sensitivity to mutant virus. (A) The number of PML NBs decreased following PML knockdown. Representative images of PML NB staining are shown for three cell lines (indicated above each column) treated with siNC or siPML. PML is indicated in red, Sp100 as green and DAPI counterstain in blue. Co-localizations are indicated in yellow. (B) siPML treatment results in decreased resistance to mutant virus. Example of a plaque assay performed in untreated IIICF/c cells or after transfection with siNC or siPML. (C) Reduction of PML NB numbers by siATRX and/or siPML. HT1080 cells were treated with siNC, siPML, siATRX or siATRX and siPML. Red staining indicates PML protein, green staining represents Sp100 and blue fluorescence is the DNA counterstain DAPI. The scale bars indicate 10 µM.

Journal of Cell Science • Supplementary information

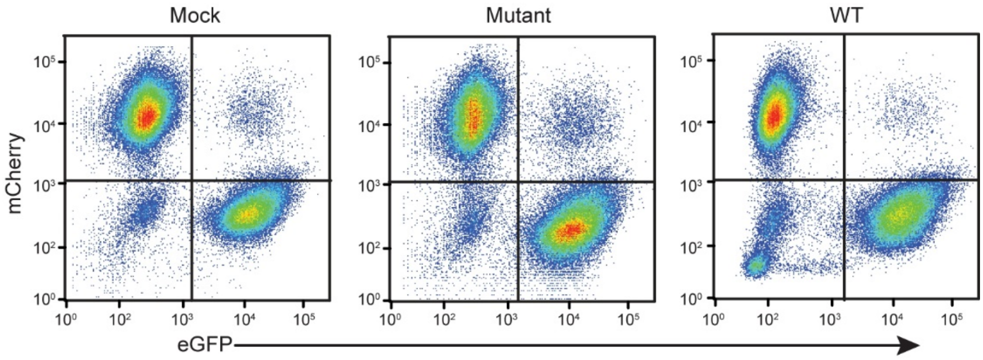

Fig S4. ATRX-negative cells are more sensitive to mutant HSV-1. Representative FACS plots of co-cultured eGFP-labeled Fre-16s primary human fibroblasts and mCherry-labeled U-2 OS ATRX-negative ALT osteosarcoma cells infected for 30 h as indicated above each plot. The double-negative and double-positive compartments presumably represent cells that have ceased expressing the marker, and ungated doublets, respectively. Data are plotted in Fig. 6C.

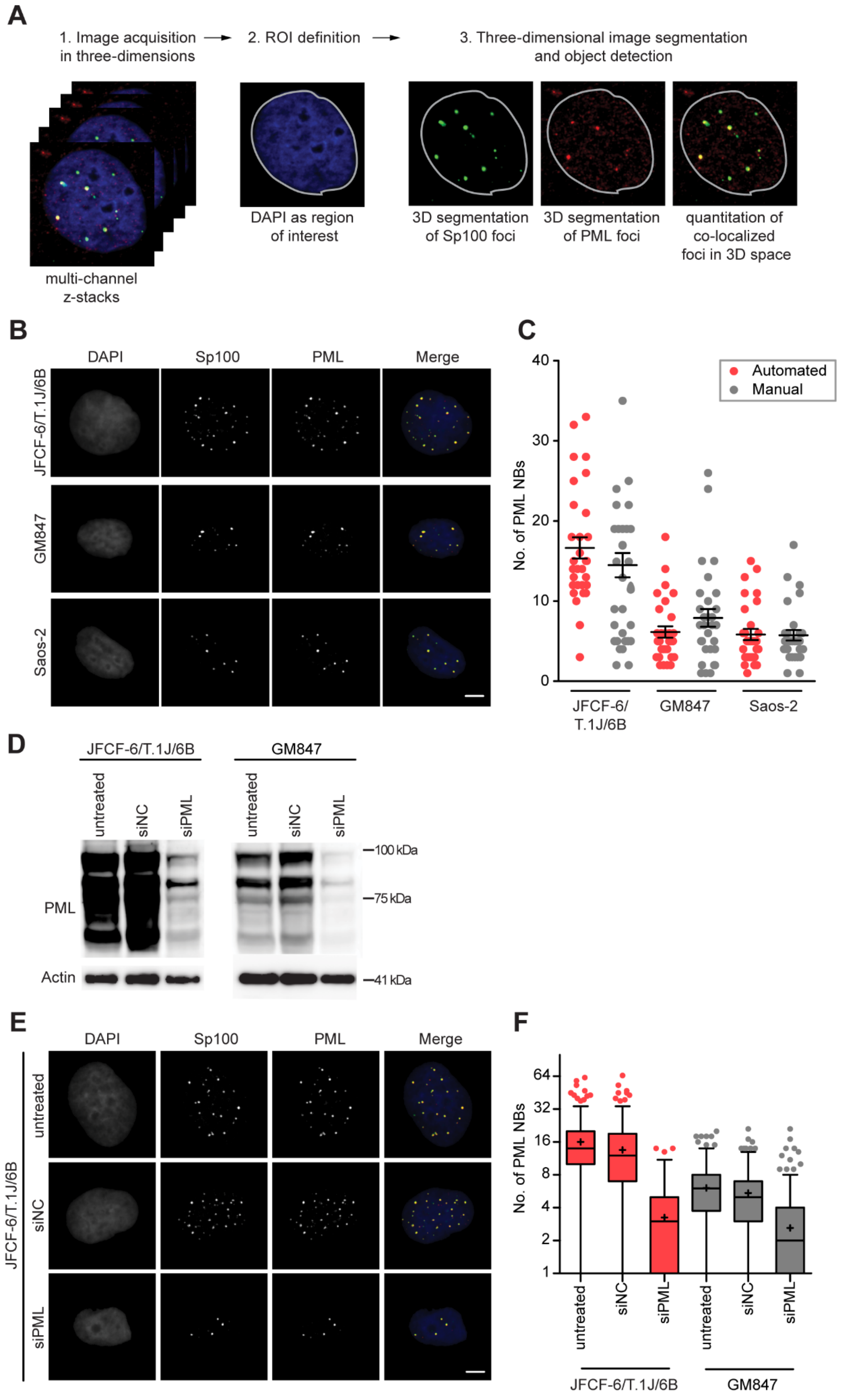

Journal of Cell Science • Supplementary information

Fig. S5. Development and optimization of high content screening for assaying PML NBs. (A) Summary of automated detection and quantitation of PML NBs. PML NBs are labeled with antibodies to PML (red) and Sp100 (green), and interphase nuclei are counterstained with DAPI (blue). Images are acquired as z-stacks and analyzed using a high-content wide-field microscope. DAPI staining is used as nuclear mask to mark the regions of interest (ROIs), Sp100 and PML foci inside this mask are identified, and then their sizes and relative intensities are quantified. Co-localized Sp100 and PML foci are identified by measuring distance between individual spots and the number of PML bodies determined as co-localized Sp100 and PML foci within 0.3 μm. Data are reported as PML NBs per nuclei. (B) Wide-field fluorescent images of JFCF-6/T.1J/6B, GM847 and Saos-2 cells labeled with antibodies against PML and Sp100 proteins, and counterstained with DAPI. (C) Quantitation of co-localizations between PML and Sp100 foci in cells from (B) completed manually, or using the automated algorithm from (A) on identical nuclei (n=30 nuclei, mean±s.e.m.). (D) Western blots of whole cell lysates from JFCF-6/T.1J/6B and GM847 cells treated with control (siNC) or siPML. (E) Corresponding immunofluorescent images of JFCF-6/T.1J/6B cells from (D) treated as indicated to the left of each row. (F) Automated quantitation of JFCF-6/T.1J/6B and GM847 cells from (D) (n=334 nuclei, displayed as a Tukey box plot with the mean indicated by “+”). The scale bars indicate 10 μM.

Table S1. Details regarding the telomere lengthening mechanism (TLM), ATRX status, immortalization method of the cell line panel, and p53 status (WT, wild-type; N, null; M, mutant; T ag, p53 inactivation by SV40 large T antigen). Note that all of the ATRX-deficient/TEL cell lines were created by experimental manipulation, whereas spontaneous ATRX deficiency is common in ALT cell lines.

Cell line	TLM	AATRX status	Method of immortalization	p53 status
U-2 OS	ALT	negative	tumor	WT
Saos-2	ALT	negative	tumor	N
SUSM-1	ALT	negative	chemical	M
IIICF/c	ALT	negative	spontaneous	N
GM847	ALT	negative	SV40 transformation	T ag
JFCF-6/T.1/P-sc2	ALT	negative	SV40 transformation	T ag
JFCF-6/T.1M	ALT	negative	SV40 transformation	T ag
JFCF-6/T.1R	ALT	negative	SV40 transformation	T ag
JFCF-6/T.1J/5H	ALT	negative	SV40 transformation	T ag
JFCF-6/T.1J/1.3C	ALT	negative	SV40 transformation	T ag
SK-LU-1	ALT	positive	tumor	M
G292	ALT	positive	tumor	M
LFS-05F-24	ALT	positive	spontaneous	N
IVG-bf/LXSN	ALT	positive	spontaneous	N
IIICF-E6E7/C4	ALT	positive	HPV	N
MeT-4A	ALT	positive	SV40 transformation	T ag
HCT116 $ATRX^{N/O}$	TEL	negative	tumor	WT
Fre80-3T-shATRX-3	TEL	negative	SV40 transformation	T ag
Fre80-3T-shATRX-7	TEL	negative	SV40 transformation	T ag
HCT116	TEL	positive	tumor	WT
HT1080	TEL	positive	tumor	WT
HT1080-6TG	TEL	positive	tumor	M
HeLa	TEL	positive	tumor	WT
A549	TEL	positive	tumor	WT
JFCF-6/T.1F	TEL	positive	SV40 transformation	T ag
JFCF-6/T.1J/6B	TEL	positive	SV40 transformation	T ag
Fre80-3T-sc2	TEL	positive	SV40 transformation	T ag
JFCF-6/T.1/P-sc1	TEL	positive	SV40 transformation	T ag
